# A dose-related miRNA panel reveals candidate tissue markers and potential mechanisms for sunset yellow-induced testicular toxicity

**DOI:** 10.29219/fnr.v70.14436

**Published:** 2026-10-26

**Authors:** Mahintaj Dara, Fatemeh Nazari, Sanaz Alaee, Zahra khodabandeh

**Affiliations:** 1 Stem Cells Technology Research Center, Shiraz University of Medical Sciences, Shiraz, Iran; 2 Department of Biology, Faculty of Science, Hakim Sabzevari University, Sabzevar, Iran; 3 Department of Reproductive Biology, School of Advanced Medical Sciences and Technologies, Shiraz University of Medical Sciences, Shiraz, Iran; 4 Netherlands Institute for Neuroscience, Royal Netherlands Academy of Arts and Sciences, Amsterdam, the Netherlands

**Keywords:** Sunset Yellow, microRNA, miR-34c, miR-122, let-7a

## Abstract

**Background:**

Sunset Yellow (SY), a widely used azo dye food additive, has raised increasing health concerns. However, its potential testicular toxicity and the underlying molecular mechanisms, particularly those involving regulatory microRNAs (miRNAs), remain poorly understood.

**Objective:**

This study aimed to investigate the effects of SY exposure on testicular histopathology and the expression patterns of key miRNAs (miR-34c, miR-122, and let-7a) in a rat model.

**Design:**

Rats were exposed to low and high doses of SY for 28 days. Testicular tissues were analyzed using Hematoxylin and Eosin (H&E) and quantitative PCR for miRNA expression. Statistical analysis was performed to assess dose-dependent effects.

**Results:**

SY exposure induced severe histopathological damage in a dose-dependent manner, including disrupted seminiferous tubule architecture, germ cell depletion, vacuolization, and interstitial cell changes. High-dose SY caused significant downregulation of miR-34c (*P* < 0.0001) and upregulation of miR-122 (16.2-fold, *P* < 0.0001), while let-7a showed alteration only at high doses. The miRNA expression changes showed a parallel association with the severity of histological impairment at the group level.

**Conclusion:**

This study demonstrates that SY induces dose-related testicular toxicity via dysregulation of miR-34c and miR-122. These candidate tissue markers are associated with testicular damage and support risk assessment and regulatory strategies, although further mechanistic and validation studies are needed.

## Popular scientific summary

- Sunset Yellow is a common food dye used in candies, soft drinks, and snacks. This study investigated its effects on the testicles of rats.
- Results showed that Sunset Yellow caused serious, dose-related damage to testicular tissue, including disrupted sperm tubes and loss of germ cells. It also strongly altered two key miRNAs: miR-34c decreased, while miR-122 increased 16-fold.
- These findings suggest that Sunset Yellow may harm male reproductive health by disrupting genetic regulators. This provides new candidate markers for food safety assessment and regulatory policy, although further validation in accessible samples is needed.

Male infertility has emerged as a pressing global health concern, affecting approximately 8–12% of couples worldwide, with male factors contributing to nearly half of all cases (1, 2). Alarmingly, recent epidemiological studies have documented a dramatic 50–60% decline in sperm counts among Western populations over the past four decades, a trend increasingly attributed to environmental factors (3). Among these potential threats, synthetic food additives have come under particular scrutiny for their possible endocrine-disrupting effects (4). This concerning backdrop has intensified research into the molecular mechanisms underlying environmental impacts on male fertility, with miRNAs emerging as critical regulators of spermatogenesis and sensitive biomarkers of reproductive health (5).

miRNAs, small non-coding RNA molecules approximately 22 nucleotides in length, have been identified as master regulators of spermatogenesis, fine-tuning gene expression at all stages of germ cell development (6, 7). Of the more than 200 miRNAs expressed in mammalian testes, three in particular – miR-34c, miR-122, and let-7a – have been shown to play indispensable roles in male fertility. miR-34c serves as a crucial supervisor of meiotic progression and spermatid maturation, primarily through its regulation of apoptosis and cell cycle proteins (8). Clinical studies have revealed its levels to be markedly reduced (by 60%) in testicular biopsies from men with non-obstructive azoospermia (9). Similarly, miR-122 acts as a metabolic guardian in the testes, modulating oxidative stress responses and maintaining the integrity of the blood-testis barrier, with its expression showing increase in cases of varicocele-associated infertility (10). Meanwhile, let-7a functions as the architect of spermatogonial stem cell dynamics, controlling the delicate balance between self-renewal and differentiation, and is notably depleted (by 80%) in Sertoli cell-only syndrome (11).

Against this background, the ubiquitous food additive Sunset Yellow (SY) (E110) has raised significant concerns (12). Consumed daily by millions through processed foods and beverages, this synthetic azo dye has demonstrated disturbing reproductive effects in animal studies, including dose-dependent reductions in sperm counts (up to 40% at 50 mg/kg/day), induction of testicular oxidative stress, and disruption of steroidogenic enzyme expression (12, 13). However, despite growing evidence linking both miRNA dysregulation and SY exposure to male infertility, a crucial knowledge gap remains regarding whether and how this common food additive might exert its reproductive effects through miRNA-mediated mechanisms.

This study represents the first comprehensive investigation into the potential impact of SY on these critical spermatogenic miRNAs. We aim to systematically characterize SY-induced alterations in miR-34c, miR-122, and let-7a expression patterns, correlate these changes with histological parameters and dose-response patterns, and establish potential causal relationships through careful dose-response analysis.

## Material and methods

### Experimental animals and study protocol

In this study, 36 male Sprague-Dawley rats weighing 40–50 grams and aged 21 days were obtained from the Shiraz University of Medical Sciences Animal Center. The animals were housed under a 12:12-h light/dark cycle at a controlled temperature of 25°C, with ad libitum access to food (standard pellet diet) and water.

The European Food Safety Authority (EFSA) has established an acceptable daily intake (ADI) of 4 mg/kg body weight/day for SY (13). For our experiment, we selected a low dose (2.5 mg/kg/day, below the ADI) and a high dose (70 mg/kg/day, substantially exceeding the ADI) (13). Notably, a study in Iran reported that 52% of restaurant food samples contained synthetic dyes, including SY, at concentrations ranging from 0.50 to 81.27 mg/kg food (14). Given these findings, we selected a low dose (2.5 mg/kg/day, below the EFSA ADI) and a high dose (70 mg/kg/day, substantially exceeding the ADI) based on previous toxicological studies (15).

For 28 consecutive days (from postnatal day 22 [P22] to day 50 [P50]), the rats received daily oral gavages. They were randomly divided into 3 groups (*n* = 12 per group): Control group: Received deionized water, Low-SY group: Administered a low dose of Sunset Yellow FCF (For Coloring Food) (2.5 mg/kg BW/day; Chemical Abstracts Service (CAS) 2783-94-0; Sigma-Aldrich, Germany), and High-SY group: Given a high dose of SY (70 mg/kg BW/day).

Sample size (*n* = 12 per group) was determined using **GPower software (version 3.1)** based on a power of 0.80, *α* = 0.05, and an effect size of 0.8 (Cohen’s *d*), derived from a pilot experiment. Animals were randomly allocated to groups using a computer-generated random number sequence (Random.org).

SY was dissolved in distilled water. All experimental protocols complied with the National Institutes of Health’s guidelines and were approved by the Institutional Animal Care and Use Committee of Shiraz University of Medical Sciences (IR.SUMS.REC.1400.453).

At the study endpoint, rats were anesthetized via intraperitoneal injection of ketamine (50 mg/kg) and xylazine (10 mg/kg; Alfasan, Netherlands) (15). Following anesthesia, euthanasia was completed by cervical dislocation. Immediately after euthanasia, the testes were excised: the right testis was flash-frozen in liquid nitrogen and stored at −80°C for RNA analysis, while the left testis was fixed in 10% neutral buffered formalin for histological examination (H&E staining).

### miRNAs extraction and molecular analysis

Total miRNA was isolated from testicular tissue using RNX-Plus reagent (Cinnagen, Iran) following the manufacturer’s protocol. For cDNA synthesis, 500 ng of extracted RNA was reverse transcribed in a 25-μL reaction volume using the RB-Micro RNA Synthesis kit (RNA Biotechnology Company, Iran) with stem-loop primers. The reaction mixture prepared according to the manufacturers. The thermal cycling protocol consisted of: 65°C for 10 min (initial denaturation), 55°C for 50 min (reverse transcription), 72°C for 20 min (enzyme inactivation), 4°C hold (performed on an ABI thermal cycler). The following primers were used for miRNA detection: miR-34c-5p (5′-AGGCAGUGUAGUUAGCUGAUUGC-3′), miR-122-5p (5′-UGGAGUGUGACAAUGGUGUUUG-3′), let-7a-5p (5′-UGAGGUAGUAGGUUGUAUAGUU-3′), and U6 snRNA as the endogenous control (forward: 5′-CTCGCTTCGGCAGCACA-3′; reverse: 5′-AACGCTTCACGAATTTGCGT-3′). Quantitative real-time PCR was conducted using a StepOne system (Applied Biosystems, Life Technologies) with the following cycling conditions: Initial denaturation: 95°C for 15 min, 40 cycles of: 95°C for 10 s (denaturation), 60°C for 45 s (annealing/extension).

U6 snRNA was selected as the reference gene because it showed stable expression across all experimental groups in our pilot experiments (Ct variation < 0.5 cycles). All samples were run in triplicate, and mean Ct values were used for subsequent analyses. Relative expression levels of miR-34c-5p, miR-122-5p, and let-7a-5p were calculated using the 2−ΔΔCt method (Livak). All statistical analyses (ANOVA and post-hoc tests) were performed using ΔCt values (normalized to U6) to ensure proper statistical assumptions.

### Histological processing and examination

Following formalin fixation, testicular tissues were processed through standard paraffin embedding protocols. Serial sections of 5 μm thickness were cut and mounted on slides. Tissue sections underwent graded ethanol dehydration (70, 80, 90%, and absolute alcohol) followed by xylene clearing and paraffin impregnation. Hematoxylin and Eosin (H&E) staining was performed according to established protocols.

Histopathological assessment was performed by two independent pathologists who were blinded to the treatment groups. Inter-observer agreement was >90%.

Microscopic evaluation was conducted using a Carl Zeiss light microscope (Meditec Inc., Jena, Germany) equipped with high-resolution differential interference contrast (DIC) optics. For qualitative assessment, 10 randomly selected, non-overlapping fields from each testicular section from 10 animals in the Control group, 11 animals in the Low-SY group, and 10 animals in the High-SY group (due to technical issues during tissue processing) were analyzed to determine tissue morphology alterations.

### Statistical evaluation

All quantitative data were analyzed using GraphPad Prism version 9 (GraphPad Software Inc., La Jolla, CA, USA). Intergroup comparisons were made using one-way Analysis of Variance (ANOVA) with Tukey’s multiple comparisons test for post-hoc analysis. Results were considered statistically significant at *P* < 0.05. Data are presented as mean ± standard error of the mean (SEM) with appropriate graphical representations.

## Results

We compared the expression levels of miR-34c, miR-122, and let-7a between the control (*n* = 12) and treated (Low-SY, *n* = 12; High-SY, *n* = 12) groups using qPCR.

### miR-34c expression analysis

qPCR analysis revealed significant downregulation of miR-34c expression in both experimental groups compared to controls. In the Low-SY group, we observed a marked reduction in miR-34c-5p expression levels (–4.2-fold, 95% CI: –5.1 to –3.3; *P* < 0.0001). Similarly, the High-SY group showed even more greater downregulation of miR-34c-5p (-8.6-fold, 95% CI: –10.2 to –7.0; *P* < 0.0001), demonstrating greater suppression at the higher dose (Fig. 1). These consistent findings across both treatment groups suggest miR-34c may play a crucial role in testicular cell apoptosis and spermatogenesis (8). The marked decrease in this regulatory miRNA was associated with impaired germ cell development and increased DNA damage in spermatogenic cells, warranting further investigation into its exact role in SY-induced testicular toxicity and male infertility (16).

**Fig. 1:**
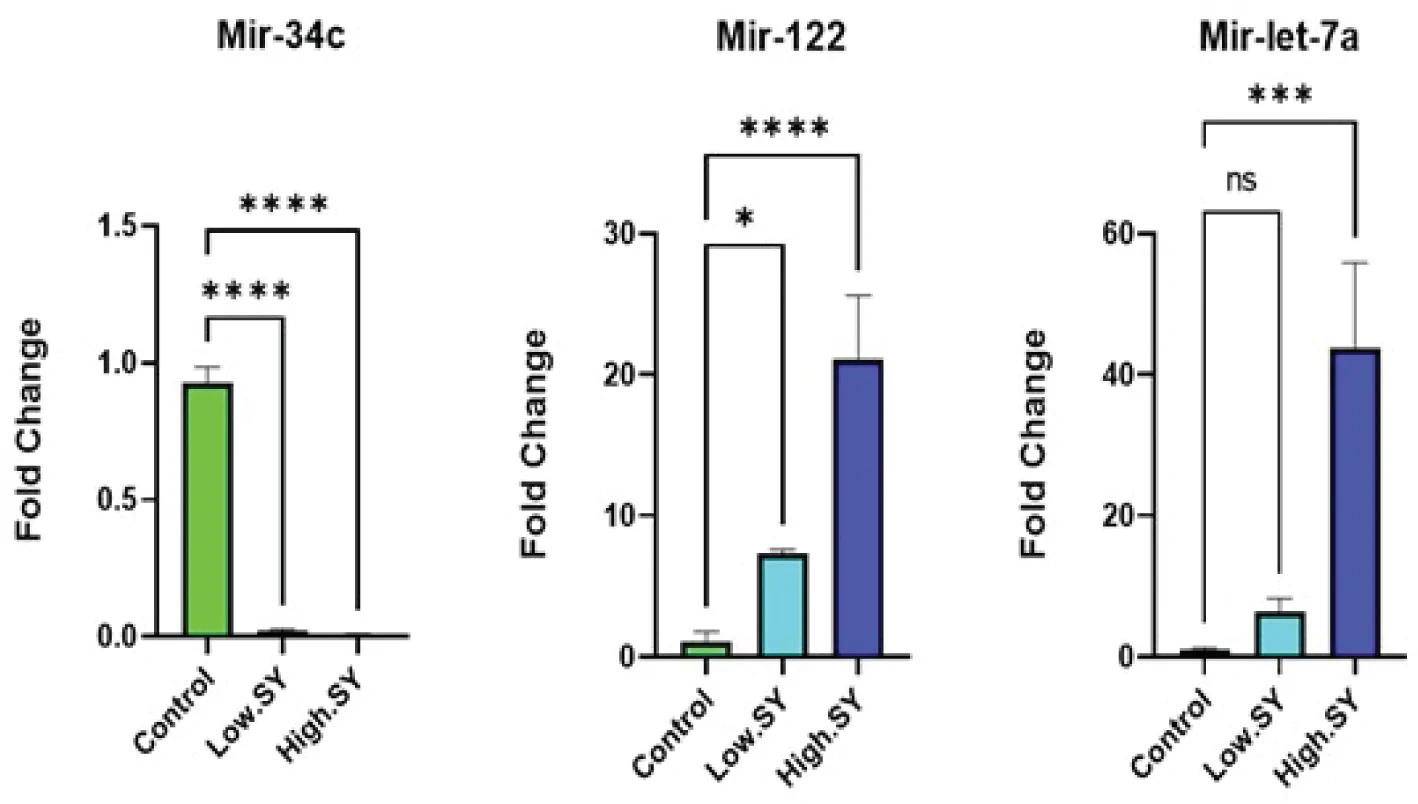
Expression analysis of microRNAs in Sunset Yellow (SY)-treated rats. Expression levels of miR-34c, miR-122, and let-7a in response to low and high doses of SY (*n* = 12). Data are mean ± standard error of the mean (SEM) (*P < 0.05, **P < 0.01, ***P < 0.001, ****P < 0.0001).

### miR-122 expression analysis

Our qPCR results demonstrated a significant, dose-dependent upregulation of miR-122 expression in response to SY treatment. The High-SY group exhibited a marked increase in miR-122 expression (+16.2-fold, 95% CI: +13.8 to +18.6; *P* < 0.0001), while the Low-SY group showed a more modest but still statistically significant elevation (+5.7-fold, 95% CI: +4.2 to +7.2; *P* = 0.0320) (Fig. 1). This consistent pattern of upregulation across both treatment groups suggests miR-122 may play an important role in the cellular response to SY exposure, with expression levels directly correlating with treatment intensity. ~~The robust response observed in the High-SY group (16.2-fold increase) compared to the Low-SY group (5.7-fold increase) indicates a clear dose-related relationship, potentially positioning miR-122 as a sensitive biomarker for SY treatment effects. These findings warrant further investigation into the precise molecular mechanisms through which SY modulates miR-122 expression and its downstream functional consequences (17).

### let-7a expression analysis

Our comprehensive evaluation of let-7a expression patterns revealed a dose-related response to SY treatment. While no significant alteration in expression levels was detected between control and Low-SY groups (*P* = 0.3433), indicating minimal impact at lower concentrations, the High-SY group exhibited a statistically significant change compared to controls (-2.8-fold, 95% CI: –3.9 to –1.7; *P* = 0.0002) (Fig. 1). These differential expression profiles suggest that let-7a responds selectively to higher SY concentrations, potentially serving as an indicator of significant exposure. The absence of expression modulation at lower doses implies either a threshold effect or the involvement of distinct regulatory mechanisms that are only activated under high-concentration conditions. This dichotomous response pattern underscores the importance of dose consideration when evaluating miRNA-based biomarkers for SY exposure, and may reflect the compound’s concentration-dependent effects on the molecular pathways governing let-7a expression.

Direct comparison between the two treatment groups revealed significant differences for all three miRNAs: miR-34c-5p (Low-SY vs. High-SY, mean difference: –4.2, 95% CI: –6.1 to –2.3; *P* < 0.01), miR-122-5p (Low-SY vs. High-SY, mean difference: +10.5, 95% CI: +7.8 to +13.2; *P* < 0.001), and let-7a-5p (Low-SY vs. High-SY, mean difference: –2.1, 95% CI: –3.4 to –0.8; *P* < 0.01). Linear trend analysis confirmed a significant dose-response relationship for miR-34c-5p (*P* < 0.0001), miR-122-5p (*P* < 0.0001), and let-7a-5p (*P* = 0.0005).

### Histopathological evaluation of SY-induced testicular toxicity

Of the 12 animals per group initially enrolled, high-quality histological sections were obtained for 10 animals in the Control group, 11 animals in the Low-SY group, and 10 animals in the High-SY group due to technical issues during tissue processing. All 12 animals per group contributed to the qPCR analysis. Comprehensive histological assessment demonstrated dose-related testicular alterations following SY exposure. Control specimens exhibited characteristic seminiferous tubule architecture with intact germinal epithelium displaying all stages of normal spermatogenesis, including properly organized spermatogonia, spermatocytes, spermatids and mature spermatozoa. In stark contrast, High-SY treatment induced severe testicular damage marked by: 1) significant tubular atrophy with epithelial disorganization, 2) substantial germ cell depletion at all developmental stages, 3) prominent luminal fibrinoid debris indicative of cellular necrosis, and, 4) marked interstitial edema with pathological vacuolization and increased interstitial cellularity. Notably, Low-SY exposure preserved testicular histoarchitecture without significant morphological deviations from controls, establishing a clear toxicity threshold (Fig. 2). These findings collectively demonstrate that while high-dose SY administration disrupts spermatogenic function through structural compromise and germ cell degeneration, low-dose exposure maintains testicular integrity, suggesting potential implications for food safety risk assessment. The distinct dose-response relationship underscores the need for further investigation into SY’s mechanisms of reproductive toxicity, particularly regarding its effects on Sertoli cell function and blood-testis barrier maintenance. The lesions described above (tubular atrophy, germ cell depletion, vacuolization, and interstitial edema) were consistently observed in the majority of High-SY animals, while they were rare or absent in the Control and Low-SY groups. Notably, the severity of histological alterations showed a parallel association with the magnitude of miRNA expression changes at the group level.

**Fig. 2:**
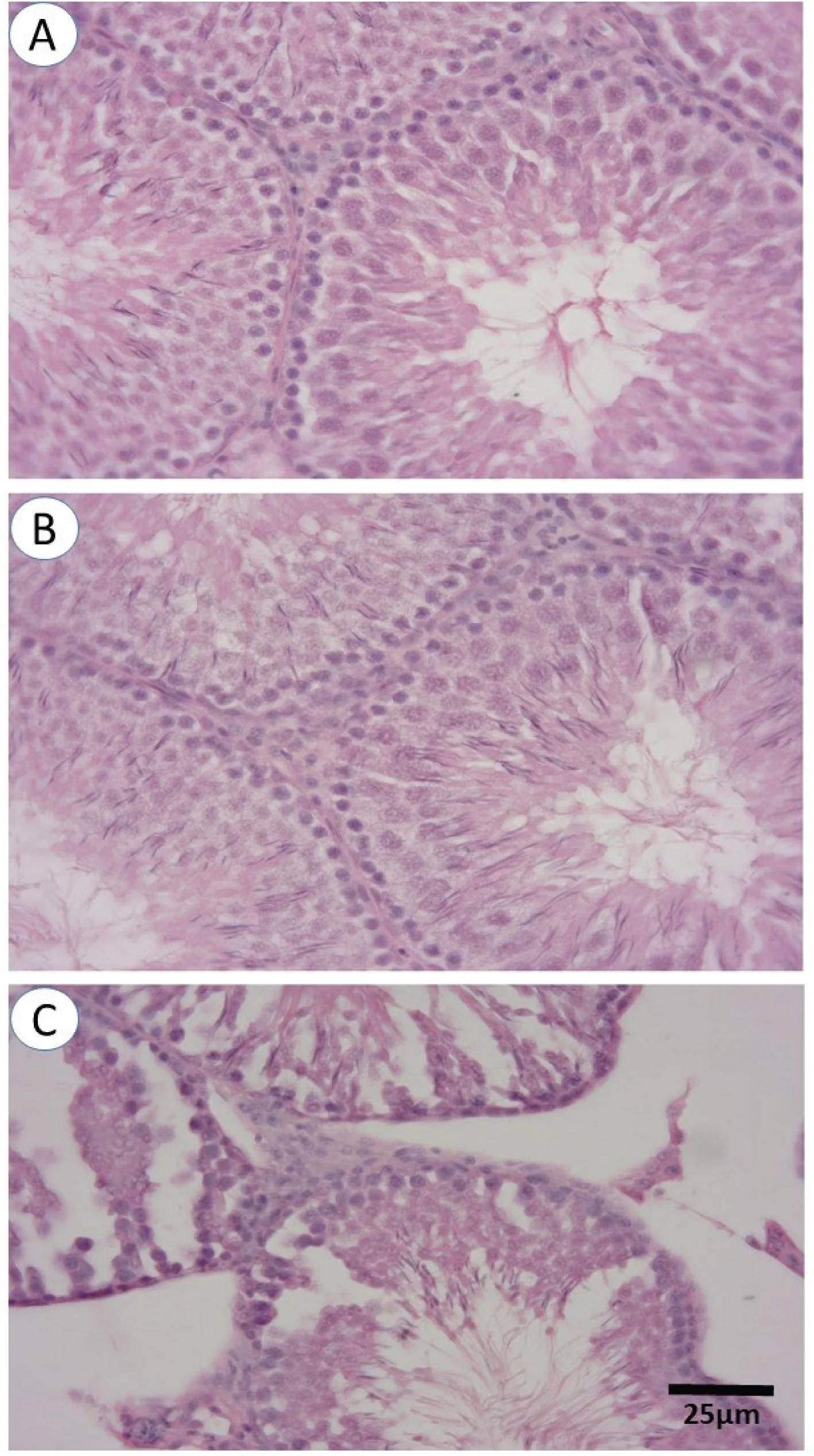
Histopathological alterations in rat testes following exposure to low and high doses of Sunset Yellow (SY). Photomicrographs of Hematoxylin and Eosin (H&E)-stained testicular sections from (A) control, (B) low-dose (LD), and (C) high-dose (HD) SY-treated groups. Scale bar = 25 µm.

## Discussion

This study provides novel insights into the molecular and histopathological effects of SY exposure through systematic analysis of three key miRNAs (miR-34c, miR-122, and let-7a) alongside detailed testicular histology. Our results demonstrate a clear dose-related response pattern across all parameters examined, revealing both potential candidate markers of exposure and suggesting possible mechanisms of testicular toxicity that warrant further investigation.

The consistent and profound downregulation of miR-34c in both Low-SY and High-SY groups (*P* < 0.0001) suggests this miRNA may serve as an indicator of SY exposure. Given its established role in regulating apoptosis and stress responses in germ cells (18), its reduced expression may be associated with disrupted spermatogenic turnover and could potentially contribute to the observed histopathological changes. The dose-dependent nature of this suppression implies SY may directly or indirectly modulate miR-34c regulatory pathways, although this interpretation is limited by the whole-tissue nature of our analysis (see below).

An important caveat regarding our miR-34c findings is that whole-tissue miRNA measurements do not distinguish between true transcriptional suppression and changes in cellular composition. Since miR-34c is highly expressed in spermatogenic cells, the observed reduction in whole-testis miR-34c levels in the High-SY group may partly reflect the loss of miR-34c-expressing germ cells rather than direct inhibition of miR-34c transcription by SY. Future studies employing in situ hybridization to localize miR-34c expression within specific testicular cell types, or using isolated germ cell populations, are needed to determine the cellular origin of these miRNA changes and to distinguish between these possibilities.

Conversely, miR-122 showed significant upregulation that closely correlated with SY dosage (5.7-fold in Low-SY, 16.2-fold in High-SY). This robust response suggests miR-122 may participate in cellular detoxification pathways, possibly through its established role in oxidative stress regulation (19). The strong dose-response relationship positions miR-122 as a promising candidate exposure biomarker worthy of further validation. However, it should be noted that the association between miRNA changes and histological findings is based on group-level parallel observations, as formal correlation analysis was not performed due to the qualitative nature of the histopathological assessment.

The dichotomous response of let-7a (no change in Low-SY vs significant alteration in High-SY) reveals a potential threshold-like pattern. This pattern mirrors our histological findings where only High-SY caused structural damage, suggesting let-7a may be part of protective mechanisms that become overwhelmed at higher doses. As let-7 family members regulate cell differentiation and proliferation (20), their dysregulation could directly impact spermatogenic efficiency.

Histopathological findings provide crucial context for the miRNA data. The preserved testicular architecture in Low-SY groups despite miRNA changes suggests compensatory mechanisms may maintain functionality below the toxicity threshold. However, High-SY-induced damage (tubular atrophy, germ cell depletion) correlates with the most severe miRNA alterations, indicating possible points of system failure. We propose several biologically plausible hypotheses that could explain our observations and should be tested in future studies:

1. Blood-testis barrier disruption: SY exposure may compromise the integrity of the blood-testis barrier, potentially exposing developing germ cells to aberrant miRNA signaling.
2. Oxidative stress induction: Reactive oxygen species generated during SY metabolism could modify the cellular microenvironment, leading to altered miRNA expression patterns.
3. Sertoli cell dysfunction: Direct toxicity to Sertoli cells may impair their crucial supportive role in spermatogenesis, subsequently disrupting miRNA-mediated germ cell communication.

We emphasize that these pathways were not directly measured in the present study and are proposed as hypotheses for future mechanistic investigations.

Collectively, our findings illuminate novel molecular mechanisms potentially involving blood-testis barrier disruption, oxidative stress, and Sertoli cell dysfunction that underpin SY-induced reproductive impairment. The compelling parallel association between miRNA alterations and histological outcomes provides a powerful framework for advancing risk assessment paradigms.

## Study limitations and future directions

This study has several limitations that should be addressed in future research. First, while our findings show strong associations, mechanistic investigations are needed to establish definitive causal relationships between SY exposure and the observed miRNA alterations. Second, expanded time-course evaluations would help delineate the temporal dynamics of these effects. Third, future studies with larger sample sizes could enhance the statistical power and generalizability of our findings. Finally, the scope of analysis should be broadened to investigate additional miRNAs within the complex regulatory network.

Additionally, our histopathological assessment was primarily qualitative, and quantitative morphometry (e.g. seminiferous tubule diameter, germinal epithelial thickness) was not systematically performed. Future studies should include quantitative histological analyses with appropriate nested statistical methods to strengthen the correlation between structural changes and molecular alterations.

Additionally, the use of a single reference gene (U6 snRNA) for miRNA normalization, while validated in our pilot experiments, may not account for all potential biological variability. Future studies should consider multiple reference genes for more robust normalization.

Additionally, our whole-tissue miRNA measurements do not distinguish between changes in gene expression due to cellular composition changes versus true transcriptional regulation. Since miR-34c is highly expressed in spermatogenic cells, the reduced levels observed in the High-SY group may partly reflect germ cell depletion rather than direct miRNA suppression. Future studies using in situ hybridization or isolated cell populations (e.g. Sertoli cells vs. germ cells) are needed to determine the cellular origin of these miRNA changes.

Despite these limitations, our findings carry significant implications for both toxicological risk assessment and future mechanistic research. The identified miRNA signatures hold considerable promise as preliminary candidate tissue markers. However, their validation as non-invasive biomarkers will require future studies in accessible biofluids such as serum or seminal plasma. Furthermore, this data contributes critically to establishing evidence-based safety thresholds for SY applications. To advance this work, future research should prioritize: pinpointing the specific cellular origins (e.g. Sertoli versus germ cells) of the dysregulated miRNAs; conducting functional validation studies using knock-in/knock-down models to confirm predicted miRNA-target pathways; and exploring potential protective interventions to mitigate SY’s adverse effects. This work establishes a critical foundation for developing miRNA-based biomarkers and paves the way for targeted preventive strategies.

Additionally, while we have proposed several mechanistic pathways (oxidative stress, BTB disruption, Sertoli cell dysfunction) to explain our findings, these were not directly measured in this study. They should therefore be considered as hypotheses for future research rather than demonstrated mechanisms.

## Conclusion

In conclusion, this study demonstrates that SY exposure induces dose-related testicular toxicity, with greater effects at higher doses, mechanistically linked to the dysregulation of key miRNAs – specifically the suppression of miR-34c, the marked upregulation of miR-122, and the threshold-dependent alteration of let-7a. These molecular signatures are strongly correlated with progressive histopathological damage, suggesting potential mechanisms of reproductive toxicity that warrant further investigation. Our findings establish a foundation for utilizing these miRNAs as preliminary candidate tissue markers for exposure monitoring and risk assessment, although further validation studies are needed. While further studies are warranted to elucidate the precise causal relationships, this work provides a critical step toward developing preventive strategies against chemical-induced testicular injury.

## Data Availability

The datasets used and/or analyzed during the current study are available from the corresponding author on reasonable request.
